# Enhancement of the local asymmetry in the hydrogen bond network of liquid water by an ultrafast electric field pulse

**DOI:** 10.1038/s41598-019-46449-5

**Published:** 2019-07-10

**Authors:** Hossam Elgabarty, Naveen Kumar Kaliannan, Thomas D. Kühne

**Affiliations:** 1 Dynamics of Condensed Matter and Center for Sustainable Systems Design, Chair of Theoretical Chemistry, University of Paderborn, Warburger Str. 100, D-33098 Paderborn, Germany; 2 Paderborn Center for Parallel Computing and Institute for Lightweight Design, University of Paderborn, Warburger Str. 100, D-33098 Paderborn, Germany

**Keywords:** Chemical physics, Density functional theory, Molecular dynamics

## Abstract

Condensed phase electron decomposition analysis based on density functional theory has recently revealed an asymmetry in the hydrogen-bond network in liquid water, in the sense that a significant population of water molecules are simultaneously donating and accepting one strong hydrogen-bond and another substantially weaker one. Here we investigate this asymmetry, as well as broader structural and energetic features of water’s hydrogen-bond network, following the application of an intense electric field square pulse that invokes the ultrafast reorientation of water molecules. We find that the necessary field-strength required to invoke an ultrafast alignment in a picosecond time window is on the order of 10^8^ Vm^−1^. The resulting orientational anisotropy imposes an experimentally measurable signature on the structure and dynamics of the hydrogen-bond network, including its asymmetry, which is strongly enhanced. The dependence of the molecular reorientation dynamics on the field-strength can be understood by relating the magnitude of the water dipole–field interaction to the rotational kinetic energy, as well as the hydrogen-bond energy.

## Introduction

Despite of extensive scientific investigations, the physical nature of fast relaxation processes within the hydrogen-bond (HB) network of water is still poorly understood^1^. This is in part due to the fact that there is no experimental technique to directly probe the dynamics of water’s HB network. The pico- to sub-picosecond lifetimes of HBs are too short for the NMR and dielectric spectroscopy time window and is only indirectly accessible by time-resolved IR spectroscopy^2^. Terahertz (THz) spectroscopy, however, based on pulsed free-electron lasers have recently achieved subpicosecond pulse durations with very high intensities^3^. Since dielectric responses in the THz region encompass water relaxations and HB vibrations at a sub-picosecond and picosecond timescale, these developments may allow the direct experimental observation of the HB dynamics^4^, as well as the dynamics induced by near-instantaneous photoexcitation or by heating with a laser pump^2,3^.

Studying the field-induced reorientation of water by means of analytic theories remains a difficult problem, which is why atomistic molecular dynamics (MD) simulations are indispensable^5–7^. However, accurate MD simulations of water are particularly challenging due to exactly the same reasons that make water unique: non-additive cooperative effects between the tetrahedrally symmetric HB centers, combined with large polarizability and dipole moment^8,9^. In fact, employing mostly empirical water models, MD simulations have indeed provided valuable mechanistic insights into these phenomena^10–14^. Yet, these studies have also highlighted the complexity of untangling the specific effects of the field from those, for instance, due to dielectric heating and surface tension^15^. The HB network is generally reported to be stable, even beyond the limits of dielectric saturation and structural breakdown, though the specific details depend on the employed water model^5,6,16^. Different empirical water potentials variably predict that a field-strength in the range 10^8^–10^10^ Vm^−1^ is required to observe the onset of dielectric saturation^5,6,10,11,15–21^. The discrepancies between the different water models can be traced back to differences in the molecular dipole moment and to the presence or absence of molecular flexibility and polarizability. Due to the fact that the predictive power of classical MD simulations critically depends on the accuracy of the employed empirical potential, which are typically parametrized to reproduce bulk water properties under field-free conditions, the transferability of these simulations is a priori uncertain. A first-principles approach such as density functional theory (DFT) based *ab-initio* MD is particularly appealing for such studies, as the interatomic forces are calculated “on-the-fly” by accurate electronic structure methods, including the effects of polarizability and cooperativity.

In this study, we employ DFT-based *ab-initio* MD to explore the structural and dynamic response of the HB network in liquid water to a 500 fs electric field pulse. The field intensity is calibrated to cause an ultrafast sub-picosecond re-orientation and alignment of the water molecules, permitting the observation of field-induced effects within a timescale that is accessible to *ab-initio* MD simulations. We start by calibrating the field intensity required to invoke such an ultrafast process and propose an intuitive explanation for the observed dependence of the re-orientation dynamics on the field-strength. Following this we investigate the structure and dynamics of the HB network. For the sake of providing a more complete picture than the one given by a mere geometric definition of the HB^22^, we employ an energy decomposition analysis based on absolutely localized molecular orbitals^23^ (ALMO-EDA, see sec:comp-data). In particular, we investigate the effect of the pulse on the recently discovered asymmetry within the HB network of liquid water^24^, which was revealed using the ALMO-EDA method^24,25^. Asymmetry in this context means that a significant population of water molecules are simultaneously donating and accepting one strong HB and another substantially weaker one^24^. This previously unknown aspect of the water HB network was shown to provide further insights in the interpretation of some of liquid water’s spectroscopic properties^26^, namely its X-ray absorption spectrum^24^, as well as its proton nuclear magnetic shielding tensor^27^. Given the already mentioned technological progress particularly with THz laser pulses, we address the question whether in the aftermath of an intense pulse there is an enhancement of this asymmetry, leading to a signature of the HB network that is experimentally measurable.

## Results

**Figure 1:**
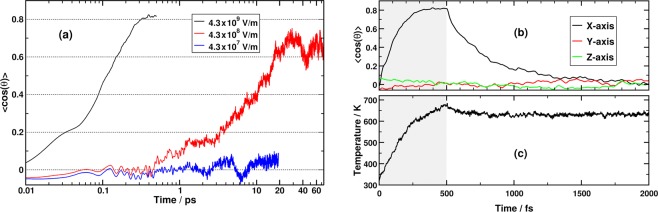
(**a**) Time evolution of the collective water re-orientation at different electric field-strengths. The angle *θ* is between the water molecular bisector and the electric field. For the strongest considered field, the average of 20 independent trajectories is plotted, while each of the other two traces is based on a single MD trajectory. (**b**) Time evolution of the average angle between the molecular bisector and the three Cartesian axes for the strongest field at 4.3 × 10^9^ Vm^−1^. The shaded region marks the duration of the electric field pulse. (**c**) Average time evolution of the temperature for the system plotted in (**b**).

In order to calibrate the strength of the applied electric field, we performed three simulations at increasing electric field intensities (Fig. 1a). The weakest electric field was set to 4.3 × 10^7^ Vm^−1^, which is comparable to the field across a biomembrane^28,29^. At this field-strength, no molecular re-orientation could be observed over a time window of 20 ps, though this does not exclude the occurrence of more subtle field-induced anisotropies^30^. Under an electric field of 4.3 × 10^8^ Vm^−1^, the water molecules are aligning their dipoles by diffusion so that within 20 ps, the average angle between the molecular bisector and the direction of the electric field stabilizes at ~50 degrees. This happens without any decrease in the hydrogen bond density, and is associated with a dielectric heating of ~10 K over 25 ps. In order to permit the observation and sampling of field-induced effects within a timescale that is accessible to *ab-initio* MD simulations, we have employed a square pulse of 4.3 × 10^9^ Vm^−1^ applied for 500 fs. This field intensity is similar in magnitude to the local field in the vicinity of electrodes^31^, ions in solution^32^, and cracks at crystal surfaces^33^. In this case, the water molecules align ballistically with the electric field and within 300 fs the average alignment angle reaches a steady state value of 37 degrees (Fig. 1b). The three field-dependent polarization responses shown in Fig. 1 closely agree with those previously reported by conventional MD simulations at the same field intensities using classical force-fields, and are consistent with the static dielectric constant of liquid water^34^ (Chapter 4).

With an ultrafast re-orientation that is much faster than any intermolecular relaxation time scale, very strong heating is observed (Fig. 1c), and the temperature continues to rise throughout the whole 500 fs duration of the pulse. In fact, this is the case even between 300–500 fs, when the average collective orientation has already stabilized, which reflects the dynamic nature of the steady-state collective orientation within this time range. After the pulse, however, the collective orientational anisotropy relaxes exponentially as expected and is complete within 1 ps. In our *ab-initio* MD simulations, which are conducted in the microncanonical ensemble, we find that the temperature drops only slightly after the pulse and then remains stable around a value of 630 K till the end of the trajectories at 2000 fs. A similar rise in temperature has also been reported previously from simulations of liquid water under the effect of a 500 fs Gaussian THz pulse with the same average electric field amplitude as the one employed here^35^.

## Discussion

The observed dependence of the collective re-orientational motion on the electric field-strength can be understood by relating the interaction energy of the latter with the molecular dipole of water. Given a field–dipole interaction that can overcome rotational thermal disorder (0.5 k_B_T along any given axis) and is thus capable of producing orientational anisotropy, the kinetics of the field-alignment process is still governed by the depth of the HB potential energy well (~8 k_B_T at room temperature^24^). Qualitatively, in the limit of a field–dipole interaction that is much stronger than this barrier, the molecules will re-orient in a ballistic manner. Even though a field that is not strong enough to overcome the bounding potential of a HB can still bias the molecular orientation and lead to orientational anisotropy, the dynamics in this case will be governed by the cooperative nature of the HB network, which enables molecular re-orientation by the simultaneous breaking and formation of HBs without the need to overcome the HB energy barrier. This is the case with the field of an intermediate strength of 4.3 × 10^8^ Vm^−1^. The same kind of cooperative dynamics is responsible for the relatively high rotational and translational diffusion coefficients of water despite of extensive hydrogen bonding^8^, which is also central in the ice to liquid phase transition, where only a little change in the HB density is accompanied by substantial changes in molecular order/disorder. As shown in Table 1, the electric field threshold to overcome the thermal rotational disorder at ambient temperature is on the order 10^8^ Vm^−1^, which is why we are considering only the strongest square pulse of 4.3 × 10^9^ Vm^−1^ from now on.

**Table 1:** Competing interactions in liquid water, which governs the re-orientation dynamics under an externally applied electric field of magnitude *E*.

	Energy/KT_298.15_
K.E. per rotational DOF^*^	0.5
Hydrogen bond energy	8.1 (ref. ^24^)
*μE* (*E* = 4.3 × 10^8^ Vm^−1^)	0.8
*μE* (*E* = 4.3 × 10^9^ Vm^−1^	8.2

The norm of the molecular dipole is taken as *μ* = *μ*_0_ + *αE*, where the field-free dipole (*μ* = 2.10 D) is the dynamic dipole as defined by^60^, whereas *α* is the polarizability of an isolated molecule at equilibrium geometry (9.785 a.u.^61^). Unlike other definitions of condensed phase molecular dipoles that rely on a partitioning of the electron density, the dynamical molecular dipole is based on the coupling of an external electric field with the molecular motion, and is thus specifically relevant for this analysis. The value of the HB energy is the ensemble-average from previous condensed phase calculations employing ALMO-EDA with the same computational setup as the one used here^24^.

^*^Degree of Freedom.

**Figure 2:**
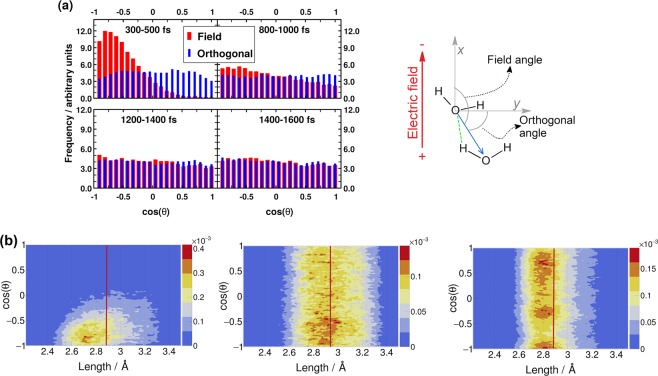
(**a**) Distribution of the angle between the HB vector and the field-direction/orthogonal field-direction (red bars/blue bars). The definitions of the HB vector and the angles are depicted in the illustration on the right. (**b**) Normalized histogram of HB lengths resolved by the angle between the electric field and the HB vector (the red bars in (**a**)). The vertical red lines indicate the average HB length in each case. Left: 300–500 fs (average = 2.88 Å), middle: 1500–2000 fs (average = 2.94 Å), right: equilibrium microcanonical distribution for comparison (average = 2.88 Å).

Figure 2a shows that during the pulse, between 300–500 fs (see time-axis in Fig. 1b,c), the field-imposed molecular orientation leads to a strong anisotropy in the HB orientation. Moreover, the better a HB is aligned with the field, the shorter it is (Fig. 2b), and that this arrangement makes charge transfer through the HB more favorable as it corresponds to a lowering in the electric potential along the HB. After the pulse, the HB orientational anisotropy decays on the same time scale as the molecular one Fig. 2b), as expected.

**Figure 3:**
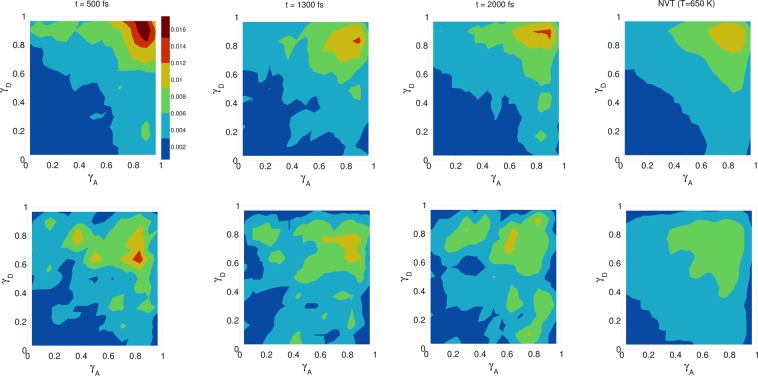
Progression of the joint distribution of the dimensionless asymmetry parameters *γ*_*A*_ and *γ*_*D*_ after the pulse (see sec:comp-data for the definition of these parameters). Time *t* = 500 fs corresponds to the end of the pulse (see time-axis in Fig. 1b,c). In the lower row only those molecules that simultaneously donate two HBs and accept two HBs, according to the employed geometric HB definition, are considered.

The influence of the pulse on the asymmetry of the HB network is shown in the upper row of Fig. 3, which depicts the joint probability distribution of the asymmetry parameters *γ*_*A*_ and *γ*_*D*_ at various times after the pulse (see sec:comp-data for definition of these parameters). /colorred To distinguish the electric-field induced effects from effects that are only due to the high temperature of the system, we also compare the joint probability distribution to that found in a field-free microcanonical trajectory simulated at an average temperature of 650 K (the two plots at the very right of Fig. 3). We see in Fig. 3 that immediately following the pulse, the probability distribution has its peak at the top right corner of the plot, where the molecules exhibit a high level of asymmetry simultaneously in the two asymmetry parameters. The asymmetry pattern in Fig. 3a is very distinct from the situation in liquid water under ambient conditions, where the largest population of molecules exhibits high asymmetry in one, but not in both asymmetry parameters. This is also distinctively different from the field-free situation in hexagonal ice, where the asymmetries in *γ*_*A*_ and *γ*_*D*_ are uncorrelated and are just a trivial consequence of the broad distribution of HB strengths that results from the phonons^25^. Comparison to field-free conditions shows that the electric field appreciably enhances the asymmetry. This enhanced asymmetry then gradually decays once the field is switched off, so that after *t* = 1.5 ps, the joint distribution has almost fully relaxed to the situation found in the high-temperature trajectory.

**Figure 4:**
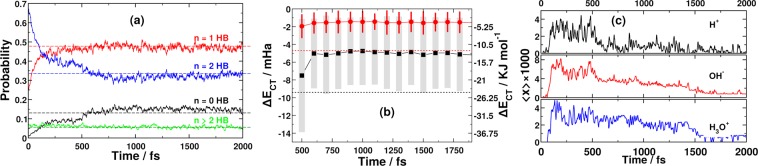
(**a**) Probability of observing an oxygen atom accepting *n* hydrogen bonds. The corresponding distribution of HB donors is analogous. The dashed lines mark the corresponding values from the high temperature field-free trajectory. (**b**) Time evolution of the HB charge transfer energy contribution. The black squares and red circles mark the strongest and the second strongest donor interactions, respectively. The dashed lines designate the corresponding energy contributions in liquid water under ambient conditions. (**c**) Average mole fraction (parts per thousand) of H^+^, OH^−^, and H_3_O^+^ residues.

Understanding the changes depicted in Fig. 3 requires an examination of the corresponding HB orientational anisotropies and the accompanying changes in the underlying structure and dynamics of the HB network. As Fig. 4a shows, the main impact of the pulse on the local HB network connectivity is a doubling in percentage of molecules that accept (or similarly, donate) a single HB (~45%), and the appearance of a sizeable population that accepts none at all (~15%). This happens at the expense of the population of molecules that accept two HBs simultaneously. Concomitantly, the HB density drastically drops throughout the first 300 fs, stabilizes between *t* = 300–500 fs, and then continues to drop again between *t* = 500–800 fs, where it stabilizes at the field-free value of 2.7 HBs per molecule (down from the initial equilibrium value of 3.7 HBs per molecule). We found the same HB density of 2.7 HB/molecule in the high-temperature trajectory (see sec:comp-data), which is comparable to previously reported HB densities under field-free and comparable thermodynamic conditions^5,36–39^. As long as one adheres to a simple geometric criterion of hydrogen bonding, our findings also agrees with previous studies that the HB network shows a remarkable stability under very strong electric fields. It should be noted however, as previously pointed out^6^, that this resilience is at least partially related to the wide tolerance of a fixed geometric definition of the HB.

In addition to the drop in HB density, the charge transfer energy (Δ*E*_*CT*_) also drops significantly after the pulse. As shown in Fig. 4b, the average Δ*E*_*CT*_ for the strongest interaction drops to around −5 m*E*_h_ and the second strongest to −1.5 m*E*_h_ (compared to −9.5 m*E*_h_ and −4.6 m*E*_h_ in ambient water, respectively). This substantial weakening of the charge transfer interaction is consistent with the increase in HB length (and drop in HB density) after the pulse (Fig. 2b), as Δ*E*_*CT*_ is known to decrease exponentially with the HB length^40^. However, during the pulse, it is obvious that because of the orientation imposed on the molecules by the field, charge transfer across most of the HBs will be enhanced. Using the average HB length under the field and the employed field intensity, the stabilization in this case amounts to ~1 m*E*_h_/meV, which is enough to render Δ*E*_*CT*_ under the field higher (in absolute value) than the corresponding value in ambient water.

**Figure 5:**
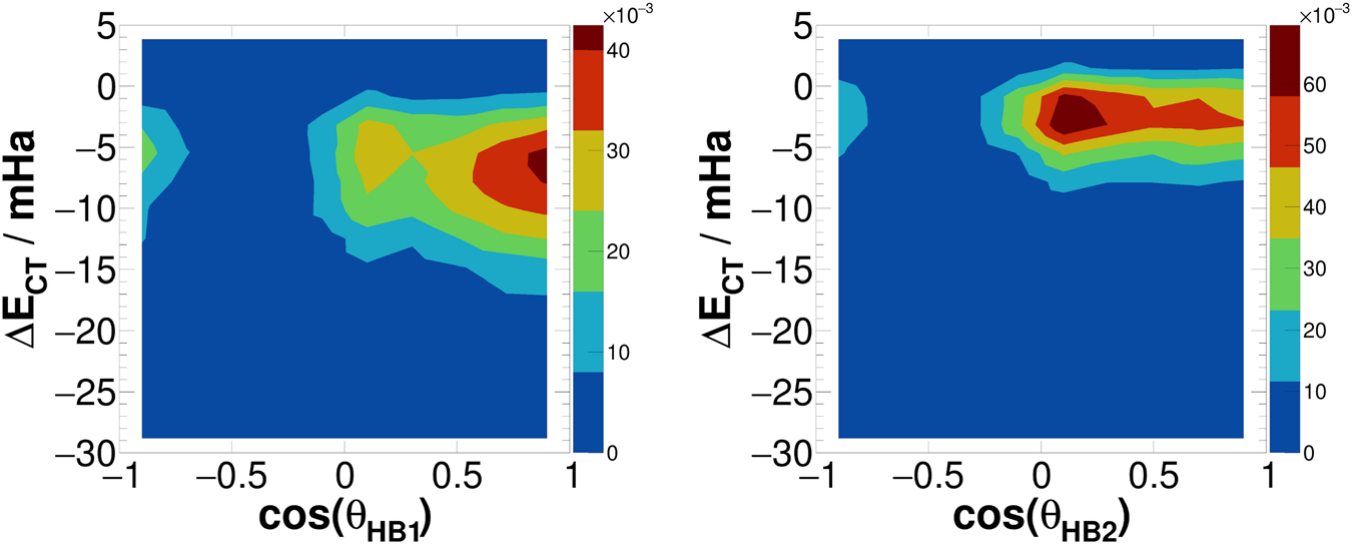
Joint distribution of the angle between a HB (HB donor to acceptor vector) and the electric field, and the corresponding ALMO-EDA charge transfer energy. In this figure only the molecules that are simultaneously accepting two HBs or simultaneously donating two HBs are considered. The left and right subfigures correspond to the strongest and second-strongest HBs, respectively. Both subfigures were calculated at *t* = 500 fs, *i.e*. immediately after the pulse.

These findings suggest that the substantially increased simultaneous asymmetry in *γ*_*A*_ and *γ*_*D*_ after the pulse is at least partly due to the increase in the population of molecules with a single donor and a single acceptor HB interaction. It should be noted however that this population of water molecules is still less than twice the population of molecules donating/accepting two HBs, meaning that the majority of the surviving HBs are still originating from the latter population. The lower row in Fig. 3 shows the joint probability distribution of the two asymmetry parameters, but only for the water molecules simultaneously engaged with two HB acceptors and two HB donors (using a geometric definition of the HB, see sec:comp-data). It is clear that in this case, there is also a significant but weaker degree of asymmetry immediately after the pulse, which however decays faster than the overall distribution in the upper row in Fig. 3. Again, the enhanced asymmtery in this population of molecules can be explained by a field-induced anisotropy in Δ*E*_*CT*_. As Fig. 5a,b show, immediately after the pulse, the strongest acceptor or donor interaction is typically pointing along the field axis, while the weaker one is more-or-less in the orthogonal plane. The molecules that are simultaneously engaged in two HBs and still do exhibit a high degree of asymmetry (*γ*_*D*_ > 0.8), are those simultaneously donating (or accepting) one HB in parallel and another one in an orientation that is more-or-less orthogonal to the field.

One further effect of the pulse that is important to mention is regarding field-induced proton currents. Previous *ab-initio* MD studies have shown that an external electric field, comparable in strength to the one employed here, leads to proton currents in water^20^. In agreement we have found that protons simultaneously hop along the field-direction in a highly concerted manner. The concerted nature of the proton hopping leads to a very small number of free ionic species (H^+^, OH^−^ and H_3_O^+^), which at any time instance is below 1% in terms of the molar ratio of these species. Within 1 ps after the pulse only one trajectory still accommodated a single dissociated water molecule (Fig. 4c).

To summarize, using DFT-based *ab-initio* MD simulations, we have shown that the non-resonant electric field-strength threshold, which induces molecular re-orientation in liquid water on the picosecond timescale, is on the order of 10^8^ Vm^−1^. It is also important to note that an ultrafast water-field alignment is still possible without a drop in the HB density, which we found to be the case under an electric field of ~4 × 10^8^ Vm^−1^ While the fact that water can align into a more ordered topology without loss of HBs is not surprising (*e.g*. in liquid to ice phase transition or while accommodating a hydrophobic solute), it is still remarkable that the requisite cooperative rearrangements of the HB network are possible in a time window of a few picoseconds. A 500 fs square pulse that is one order of magnitude stronger than this threshold causes very fast (~300 fs) alignment of the molecular dipoles and also leads to orientational anisotropies within HB lengths and strengths, as well as pair-wise charge transfer energy contributions (Δ*E*_*CT*_) as quantified by ALMO-EDA. These orientational anisotropies, together with the observed two-fold increase in the population of molecules that donate or accept a single HB, lead to an enhancement in the innate asymmetry in the HB network that survives on a picosecond timescale. Intense ultrafast electric field pulses are experimentally feasible^41^, and THz laser pulses are approaching this intensity range with recently available free electron laser technology^42,43^, opening the door for quantitative experimental investigation of these phenomena.

## Methods

### MD simulations

Ab-initio MD simulations of a periodic cubic cell with 128 water molecules were performed at constant energy and ambient density (0.9966 g cm^−3^) using the second generation Car–Parrinello method of Kühne and coworkers^44,45^. The energy and forces were computed using the mixed Gaussian-plane waves approach^46^, where the Kohn–Sham orbitals were represented by an accurate triple-*ζ* basis set with two sets of polarization functions (TZV2P)^47^, whereas a plane-waves with cutoff of 400 Ry were used to represent the charge density. The BLYP exchange-correlation functional plus a damped interatomic potential to account for van der Waals interactions (Grimme-D3^48^) was employed. Previous works have shown that this set-up provides a realistic description of many important structural, dynamical and spectroscopic characteristics of liquid water, including the partial pair correlation functions, self-diffusion and viscosity coefficients, HB lifetime, NMR shieldings, and vibrational spectra^22,24,25,27,49,50^. As we report here, the polarization response that we observe matches the response expected from the static dielectric constant of water^34^ (Chapter 4). Previous work has also suggested that this setup can reproduce the dielectric permittivity of liquid water^51^, even though the exact value of the permittivity is not known, as calculating the dielectric constant from MD simulations requires long simulation times of several nanoseconds^34,52,53^. For starting the production MD runs, the system was equilibrated for 30 ps before 20 decorrelated snapshots were extracted separated by 2 ps. Each snapshot was then used to start an individual ab-initio MD trajectory under the effect of a static electric field pulse. We used the Berry phase approach to ensure a proper description of the field under periodic boundary conditions^54–56^. A square electric field pulse pointing along the positive x-axis (*i.e*. a positive test charge would follow this direction) was applied for a duration of 500 fs, and then each trajectory was continued for further 1.5 ps after the pulse. No thermostat was employed during the production runs and all the results reported here are averaged over all the 20 non-equilibrium trajectories. For comparison to high-temperature field-free conditions, we have also simulated the same water box for 30 ps under micorcanonical constraints at an average temperature of 650 K, discarding the first 5 ps from the analysis. All computations were performed using the Quickstep module of the CP2K suite of programmes^57^. For defining a HB we used a simple geometric criterion (3.5 Å and 30 degrees)^58^.

### ALMO-EDA

ALMO-EDA proceeds by first filtering out frozen electrostatic and polarization effects from the total many-body intermolecular binding energy, and then splitting the remaining electron transfer (*i.e*. covalent) component into two-body terms, Δ*E*_*CT*_, each corresponding to an individual HB^23^. These two-body terms are obtained self-consistently under fully periodic boundary conditions and include cooperativity effects^23,24^, which are essential for a correct description of the HB network in liquid water^49,59^. The asymmetry between the two strongest HB donor interactions involving a particular water molecule can be assessed by a dimensionless asymmetry parameter

1 $${\gamma }_{D}=1-\frac{{\rm{\Delta }}{E}_{A\to {B}^{2nd}}}{{\rm{\Delta }}{E}_{A\to {B}^{1st}}},$$

where the Δ*E* terms are the two strongest two-body donor (involving charge transfer from molecule A to B) interaction energies^24^. A similar asymmetry parameter for acceptor interactions *γ*_*A*_, is also defined. In liquid water under ambient conditions, the joint distribution of *γ*_*A*_ and *γ*_*D*_ reveals that there is a significant population of molecules with both parameters close to unity^24,25^. All ALMO-EDA calculations in this work were performed using the same computational setup as in ref. ^24,25,27^ using the ALMO-EDA implementation in CP2K^23^.
